# Syntheses and crystal structures of the ethanol, acetonitrile and diethyl ether Werner clathrates bis­(iso­thio­cyanato-κ*N*)tetra­kis­(3-methyl­pyridine-κ*N*)nickel(II)

**DOI:** 10.1107/S2056989022008891

**Published:** 2022-09-08

**Authors:** Christoph Krebs, Inke Jess, Christian Näther

**Affiliations:** a Institut für Anorganische Chemie, Universität Kiel, Max-Eyth-Str. 2, 24118 Kiel, Germany; University of Aberdeen, Scotland

**Keywords:** nickel(II)thio­cyanate, 3-methyl­pyridine, clathrates, crystal structure

## Abstract

The reaction of nickel(II)thio­cyanate with 3-methyl­pyridine (3-picoline; C_6_H_7_N) in different solvents leads to the formation of crystals of bis­(iso­thio­cyanato-κ*N*)tetra­kis­(3-methyl­pyridine-κ*N*)nickel(II) as the ethanol disolvate, [Ni(NCS)_2_(C_6_H_7_N)_4_]·2C_2_H_5_OH (**1**), the acetonitrile disolvate, [Ni(NCS)_2_(C_6_H_7_N)_4_]·2CH_3_CN (**2**), and the diethyl ether monosolvate, [Ni(NCS)_2_(C_6_H_7_N)_4_]·C_4_H_10_O (**3**). The crystal structures of these compounds consist of Ni^II^ cations coordinated by two N-bonded thio­cyanate anions and four 3-methyl­pyridine ligands to generate NiN_6_ octa­hedra with the thio­cyanate groups in a *trans* orientation. In compounds **1** and **2** these complexes are located on centers of inversion, whereas in compound **3**, they occupy general positions. In the crystal structures, the complexes are packed in such a way that cavities are formed in which the solvent mol­ecules are located. Compounds **1** and **2** are isotypic, which is not the case for compound **3**. In compounds **1** and **2** the solvate mol­ecules are disordered, whereas they are fully ordered in compound **3**. Disorder is also observed for one of the 3-methyl­pyridine ligands in compound **2**. Powder X-ray diffraction and IR measurements show that at room temperature all compounds decompose almost immediately into the same phase, as a result of the loss of the solvent mol­ecules.

## Abstract 2

The crystal structures of the title compounds consist of discrete octa­hedral complexes that are arranged in such a way that cavities are formed in which the solvate mol­ecules are located.

## 1. Chemical context

The synthesis and structural characterization of new compounds is still an important topic in coordination chemistry, because some of them might have the potential for future applications such as magnetic behavior. In this context, coord­ination compounds in which the cations are linked by small-sized anionic ligands into networks of different dimensionality are of special inter­est. Therefore, many compounds based on, for example, cyanide or azide ligands have been reported in the literature. Magnetic exchange can also be mediated by thio­cyanate anions and this is one reason why we and others have been inter­ested in this class of compounds for many years (Mautner *et al.*, 2018 ▸, Rams *et al.*, 2020 ▸, Böhme *et al.*, 2020 ▸). Regarding this, compounds are of inter­est in which the paramagnetic metal cations are linked by thio­cyanate anions into chains or layers (Werner *et al.*, 2014 ▸, 2015*a* ▸,*b* ▸; Suckert *et al.*, 2016 ▸). In contrast to azides or cyanides, the synthesis of thio­cyanates with bridging coordination is more difficult to achieve, because metal cations such as Mn^II^, Fe^II^, Co^II^ and Ni^II^ are less chalcophilic and therefore prefer a terminal N coordination. Nevertheless, a large number of compounds with μ-1,3-bridging thio­cyanate anions have been reported in recent years (Mautner *et al.*, 2018 ▸ and Werner *et al*., 2015*a* ▸,*b* ▸). 
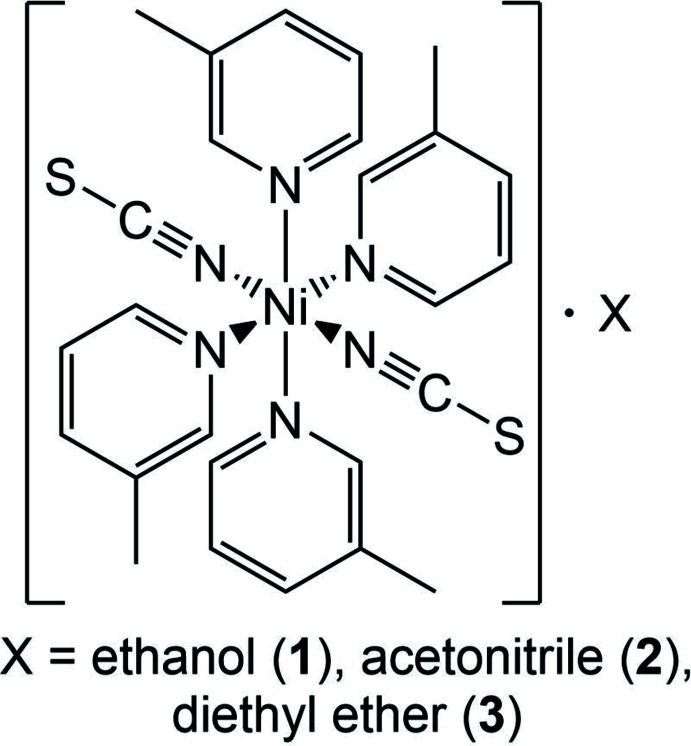


In our own investigations, we are particularly inter­ested in the influence of the neutral co-ligand on the chemical reactivity, the crystal structure and the magnetic properties of thio­cyanate coordination polymers of 3*d* metal cations. In most cases, we used pyridine derivatives that are substituted in the 4-position as co-ligands, but recently we also became inter­ested in such ligands where the substitutent is located in the 3-position, including 3-methyl­pyridine (also called 3-picoline), C_6_H_7_N. With Co(NCS)_2_, two discrete complexes with the composition Co(NCS)_2_(C_6_H_7_N)_4_ (refcodes EYAROM and EYAROM01; Boeckmann *et al.*, 2011 ▸ and Małecki *et al.*, 2012 ▸) and Co(NCS)_2_(C_6_H_7_N)_2_(H_2_O)_2_ (EYAREC; Boeckmann *et al.*, 2011 ▸) are deposited in the Cambridge Structural Database, in which the cobalt cations are octa­hedrally coordinated by two terminal N-bonded thio­cyanate anions and four 3-methyl­pyridine in the former compound and two 3-methyl­pyridine and two water ligands in the latter. Upon heating, these complexes lose half of their co-ligands and transform into Co(NCS)_2_(C_6_H_7_N)_2_ (EYARIG; Boeckmann *et al.*, 2011 ▸) before a decomposition into Co(NCS)_2_ is observed. Surprisingly, in contrast to most other compounds with pyridine derivatives substituted in the 4-position where chains or layers are formed, in this compound the Co^II^ cations are tetra­hedrally coordinated by two terminal N-bonded thio­cyanate anions and two 3-methyl­pyridine co-ligands, forming discrete complexes.

Most compounds with 3-methyl­pyridine as co-ligand are reported with Ni(NCS)_2_, but surprisingly in none of them are the Ni^II^ cations linked by the thio­cyanate anions. This includes, for example, Ni(NCS)_2_(C_6_H_7_N)_2_(H_2_O)_2_ (MEGCEH; Tan *et al.*, 2006 ▸), which is isotypic to its cobalt analog. Moreover, a number of compounds consist of discrete complexes with the general composition Ni(NCS)_2_(C_6_H_7_N)_4_ in which the Ni^II^ cations are octa­hedrally coordinated by two terminal N-bonded thio­cyanate anions as well as by four 3-methyl­pyridine co-ligands. In all of these compounds, the discrete complexes are packed in such a way that cavities are formed, in which additional solvate mol­ecules are embedded. Altogether, three different structure types are observed. The mono-di­chloro­methane (Laylus, Pang *et al.*, 1992 ▸), mono-tri­chloro­methane (CIVJEW and CIFJEW01; Nassimbeni *et al.*, 1984 ▸, 1986 ▸), mono-tetra­chloro­methane, mono-di­bromo­dichloro­methane and mono-2,2-di­chloro­propane clathrates (JICMIR, LAYLAY and LAYLEC; Pang *et al.*, 1990 ▸, 1992 ▸) crystallize in the ortho­rhom­bic space group *Fddd*. If two mol­ecules of tri­chloro­methane are incorporated, the clathrate crystallizes with triclinic symmetry in space group *P* 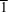 (LAYLOM; Pang *et al.*, 1992 ▸) and the bis­(di­chloro­methane) clathrate crystallizes in the monoclinic space group *C*2/*c* (LAYLIG; Pang *et al.*, 1992 ▸). It is noted that the two latter unit cells are crystallographically unrelated. The formation of these clathrates for such simple nickel complexes is surprising because this is not observed in practically all other complexes with Ni(NCS)_2_ and pyridine derivatives as co-ligands. However, it might be traced back to the fact that all of these solvents are non-polar and cannot coordinate to Ni^II^ cations to form, for example, solvato octa­hedral complexes with the composition Ni(NCS)_2_(C_6_H_7_N)_2_(*L*)_2_ (*L* = co-ligand).

Based on these assumptions, we tried to prepare additional compounds based on Ni(NCS)_2_ and 3-methyl­pyridine as co-ligand, for which we used diethyl ether, ethanol and aceto­nitrile as solvents. All of them can coordinate to Ni^II^ cations, which might lead to solvato complexes that afterwards might be transformed into the desired compounds with a bridging coordination by thermal decomposition. On the other hand, they are not very strong donor ligands, which means that compounds with a bridging coordination of the anionic ligands might form directly. With all three solvents, suitable crystals were obtained, which were characterized by single-crystal X-ray diffraction. Structure analysis reveals that even in this case, clathrates with the composition Ni(NCS)_2_(C_6_H_7_N)_4_ · 2 ethanol (**1**), Ni(NCS)_2_(C_6_H_7_N)_4_ · 2 aceto­nitrile (**2**) and Ni(NCS)_2_(C_6_H_7_N)_4_ · diethyl ether (**3**) have formed, which crystallize in two different structure types, with compounds **1** and **2** isotypic to the bis­(di­chloro­methane) clathrate reported by Pang *et al.* (1992 ▸). Unfortunately, all of these compounds lose their solvents almost immediately at room temperature and X-ray powder diffraction shows that the same crystalline phase is obtained (Fig. S1 in the supporting information). In their IR spectra, the CN stretching vibration is observed at 2074 cm^−1^, indicating that the anionic ligands are still terminally N-bonded (Fig. S2). Therefore, one can assume that a solvent-free compound with the composition Ni(NCS)_2_(C_6_H_7_N)_4_ has formed, that still consists of discrete complexes and for which the crystal structure is unknown.

## 2. Structural commentary

**Figure 1:**
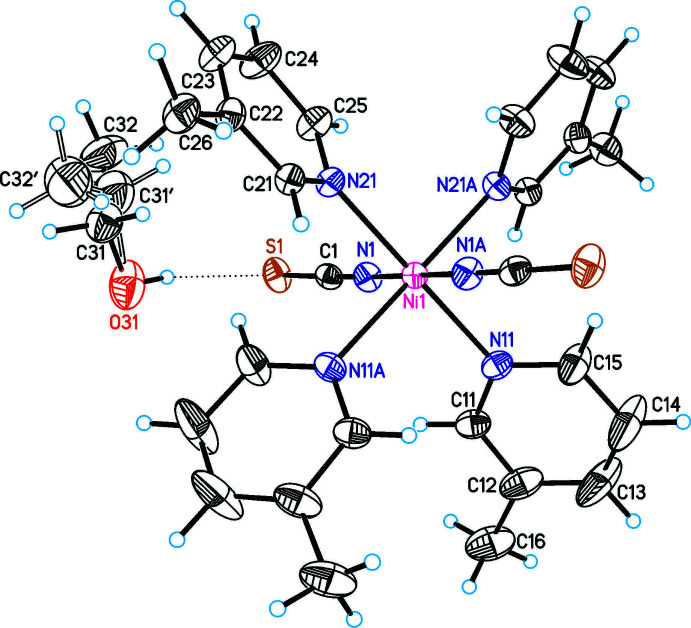
The mol­ecular structure of compound **1** with labeling and displacement ellipsoids drawn at the 50% probability level. Symmetry code: (A) −*x* + 1, *y*, −*z* + 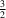 .

**Figure 3:**
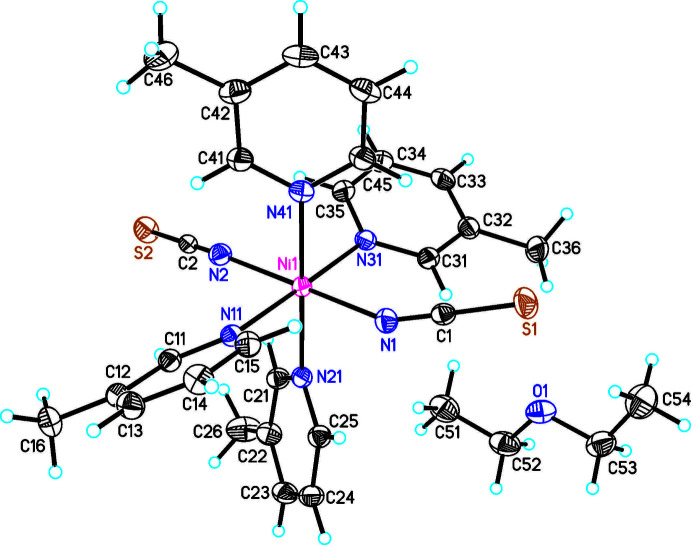
The mol­ecular structure of compound **3** with labeling and displacement ellipsoids drawn at the 50% probability level.

The asymmetric units of Ni(NCS)_2_(C_6_H_7_N)_4_ · 2 ethanol (**1**) and Ni(NCS)_2_(C_6_H_7_N)_4_ · 2 aceto­nitrile (**2**) consist of half of an Ni^II^ cation that is located on a center of inversion, one thio­cyanate anion and two 3-methyl­pyridine ligands as well as one ethanol (**1**) and one aceto­nitrile (**2**) solvate mol­ecules in general positions (Figs. 1 ▸ and 2 ▸). The asymmetric unit in Ni(NCS)_2_(C_6_H_7_N)_4_ · diethyl ether (**3**) consists of one Ni^II^ cation, two thio­cyanate anions, four 3-methyl­pyridine ligands and one diethyl ether solvate mol­ecule that occupy general positions (Fig. 3 ▸). In compounds **1** and **2**, the solvate mol­ecules are disordered and were refined using a split model (see *Refinement*), whereas in compound **3** they are fully ordered. The ethanol and aceto­nitrile solvates **1** and **2** crystallize in the monoclinic *C*-centered space group *C*2/*c* and are isotypic to the bis­(di­chloro­methane) clathrate reported by Pang *et al.* (1992 ▸). Compound **3** crystallizes in space group *P*2_1_/*n* and its structure type is different from that of the solvates of Ni(NCS)_2_(C_6_H_7_N)_4_ already reported in the literature (see *Chemical Context*).

In all three compounds the nickel(II) cations are octa­hedrally coordinated by two terminal N-bonded thio­cyanate anions and four 3-methyl­pyridine co-ligands, forming discrete complexes. In compound **1** and **2** the discrete complexes are located on centers of inversion, whereas in compound **3** the complexes are located in general positions. The Ni—N bond lengths are comparable in all three compounds (Tables 1 ▸–3 ▸ ▸) and from the bonding angles, it is obvious that all octa­hedra are slightly distorted (see supporting information). This is reflected in the octa­hedral angle variance and the mean octa­hedral quadratic elongation calculated by the method of Robinson *et al.* (1971 ▸), which amount to 0.0857°^2^ and 1.0004, respectively, for compound **1**, 0.3299°^2^ and 1.0006 for compound **2** and 1.0694°^2^ and 1.0010 for compound **3**.

## 3. Supra­molecular features

**Figure 4:**
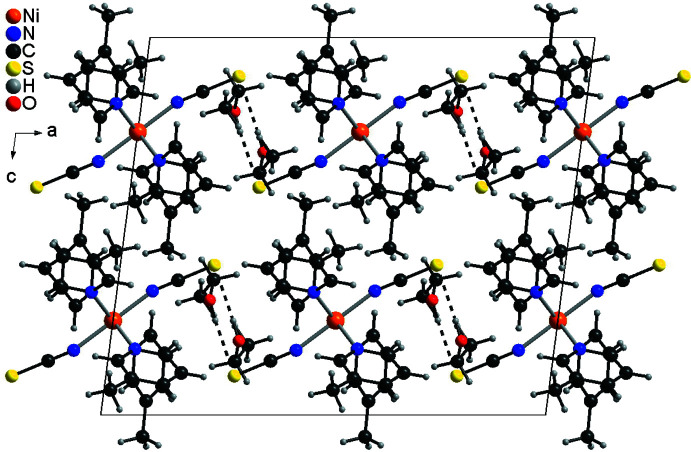
Crystal structure of compound **1** as a representative with view along the crystallographic *b*-axis and inter­molecular O—H⋯S hydrogen bonds shown as dashed lines.

**Figure 5:**
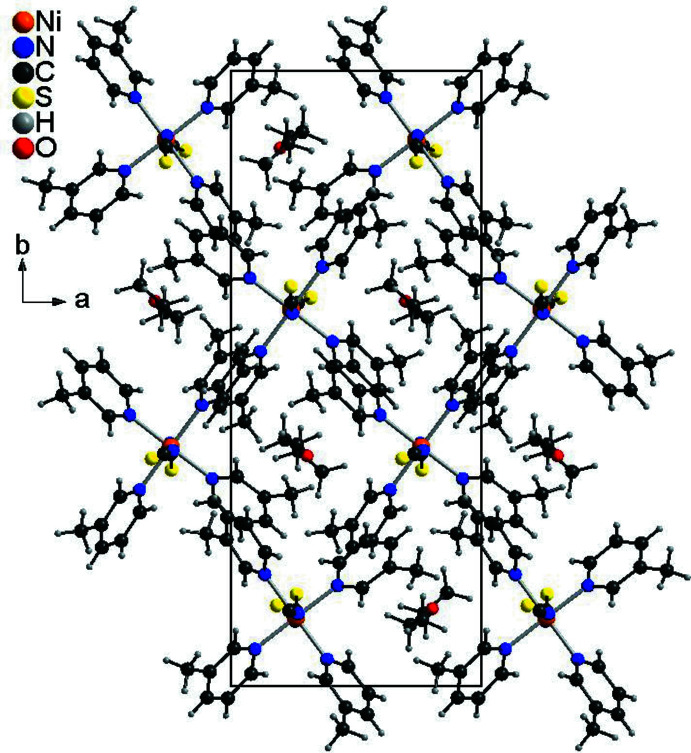
Crystal structure of compound **3** with view along the crystallographic *c*-axis.

In the crystal structures, the Ni(NCS)_2_(C_6_H_7_N)_4_ complexes are packed in such a way that cavities are formed, in which the solvate mol­ecules are embedded (Figs. 4 ▸ and 5 ▸). In compound **1**, both ethanol mol­ecules are linked to the complex by O—H⋯S hydrogen bonding between the hydroxyl hydrogen atom of the ethanol mol­ecule and the thio­cyanate S atom (Fig. 4 ▸). The H⋯S distance amounts to 2.464 (4) Å and the O—H⋯S angle to 172 (2)°, which indicates that this is a strong inter­action (Table 4 ▸). There is one additional inter­molecular contact between a pyridine H atom and the ethanol O atom, but the distance and geometry of this contact shows that this should be only a very weak inter­action (Table 4 ▸). In the isotypic compound **2**, no pronounced inter­molecular inter­actions are observed and the packing seems to be dominated by van der Waals inter­actions. This is similar in the diethyl ether solvate **3**, where the complexes are arranged in stacks along the *c*-axis direction (Fig. 5 ▸). For all compounds, the void spaces occupied by the solvate mol­ecules were calculated, leading to values of 221 Å^3^ (6.5% of the unit-cell volume) for **1**, 162 Å^3^ (4.8%) for **2** and 165 Å^3^ (5.1%) for **3**. The higher value for compound **1** might be traced back to the inter­molecular hydrogen bonding.

## 4. Database survey

Several thio­cyanate compounds with transition metal cations and 3-methyl­pyridine as co-ligand are reported in the Cambridge Structure Database CSD (version 5.43, last update November 2021; Groom *et al.*, 2016 ▸), including the Co and Ni compounds mentioned above.

With Cd(NCS)_2_, one compound with the composition Cd(NCS)_2_(C_6_H_7_N)_2_ (FIYGUP; Taniguchi *et al.*, 1987 ▸) is reported, in which the Cd^II^ cations are octa­hedrally coordinated and linked by pairs of thio­cyanate anions into chains. With copper, discrete complexes with the composition Cu(NCS)_2_(C_6_H_7_N)_2_ (ABOTET; Handy *et al.*, 2017 ▸) and Cu(NCS)_2_(C_6_H_7_N)_3_ (VEPBAT; Kabešová & Kožíšková, 1989 ▸) are reported. There is also one chain compound with the composition Cu(NCS)_2_(C_6_H_7_N)_2_ (CUHBEM; Healy *et al.*, 1984 ▸), in which the copper cations are tetra­hedrally coord­inated. With Zn(NCS)_2_, the discrete complex Zn(NCS)_2_(C_6_H_7_N)_2_ with a tetra­hedral structure is found (ETUSAO; Boeckmann & Näther, 2011 ▸), which is isotypic to Co(NCS)_2_(C_6_H_7_N)_2_. With Mn^II^ and Fe^II^, two discrete complexes with the composition *M*(NCS)_2_(C_6_H_7_N)_4_ (*M* = Mn, Fe) are reported (Ceglarska *et al.*, 2022 ▸). Additionally there is also a mixed-metal compound with manganese and mercury with the composition *catena*-[tetra­kis­(thio­cyanato)­bis­(3-meth­yl­pyridine)­manganese-mercury] (NAQYOW; Małecki, 2017 ▸).

## 5. Synthesis and crystallization

**Synthesis**

3-Methyl­pyridine was purchased from Alfa Aesar. Ni(NCS)_2_ was purchased from Santa Cruz Biotechnology. Aceto­nitrile was dried over CaH_2_ and ethanol over sodium before use.

Ni(NCS)_2_(C_6_H_7_N)_4_ · 2 ethanol (**1**): 0.25 mmol Ni(NCS)_2_ (43.7 mg) and 2.5 mmol 3-methyl­pyridine (243 µl) were added to 1.5 ml of ethanol and stored under hydro­thermal conditions at 403 K to form light-purple single crystals.

Ni(NCS)_2_(C_6_H_7_N)_4_ · 2 aceto­nitrile (**2**): To synthesize single crystals suitable for single-crystal X-ray analysis, 0.25 mmol of Ni(NCS)_2_ (43.7 mg) and 2.5 mmol of 3-methyl­pyridine (243 µl) were combined in a snap-cap vial and 1.5 ml of aceto­nitrile were added. After two days at room temperature, light-purple blocks were obtained.

Ni(NCS)_2_(C_6_H_7_N)_4_ · di­ethyl­ether (**3**): In a mixture of diethyl ether and H_2_O, 0.25 mmol of Ni(NCS)_2_ (43.7 mg) and 2.5 mmol of 3-methyl­pyridine (243 µl) were added. Single crystals in the form of light-purple blocks were obtained after heating the reaction mixture to 353 K and storing it at this temperature for two days.

## 6. Refinement

Crystal data, data collection and structure refinement details are summarized in Table 5 ▸. The C-bound H atoms were positioned with idealized geometry (methyl H atoms allowed to rotate but not to tip) and were refined isotropically with *U*_iso_(H) = 1.2 *U*_eq_(C) (1.5 for methyl H atoms) using a riding model.

## Supplementary Material

Crystal structure: contains datablock(s) 1, 2, 3, global. DOI: 10.1107/S2056989022008891/hb8036sup1.cif

Structure factors: contains datablock(s) 1. DOI: 10.1107/S2056989022008891/hb80361sup2.hkl

Structure factors: contains datablock(s) 2. DOI: 10.1107/S2056989022008891/hb80362sup3.hkl

Structure factors: contains datablock(s) 3. DOI: 10.1107/S2056989022008891/hb80363sup4.hkl

Experimental X-ray powder patterns of the residues obtained by storing the compounds 1 (A), 2 (B) and 3 (C) for one hour at room-temperature. DOI: 10.1107/S2056989022008891/hb8036sup5.png

IR spectra of the residues obtained by storing the compounds 1 (A), 2 (B) and 3 (C) for one hour at room-temperature. The value of the CN stretching vibrations is given. DOI: 10.1107/S2056989022008891/hb8036sup6.png

CCDC references: 2205563, 2205564, 2205565

Additional supporting information:  crystallographic information; 3D view; checkCIF report

## Figures and Tables

**Figure 2:**
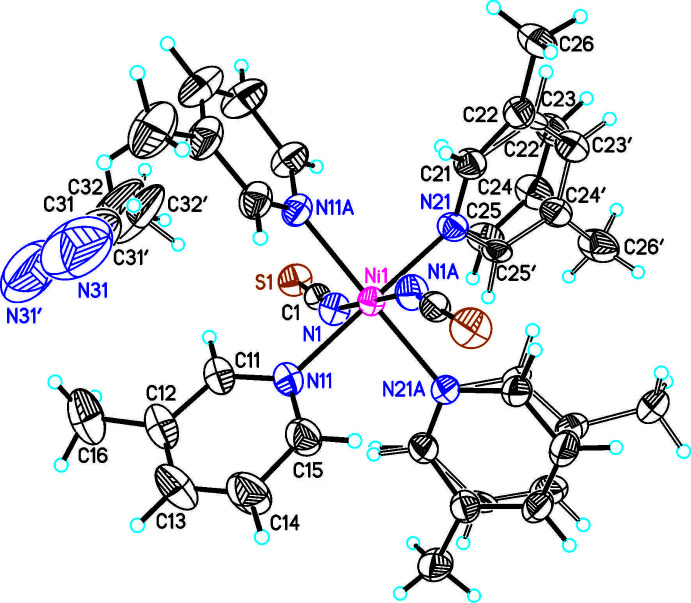
The mol­ecular structure of compound **2** with labeling and displacement ellipsoids drawn at the 50% probability level. Symmetry code: (A) −*x* + 1, *y*, −*z* + 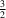 .

**Table 1:** Selected bond lengths (Å) for **1** [Chem scheme1]

Ni1—N1	2.0597 (13)	Ni1—N21	2.1200 (11)
Ni1—N11	2.1196 (12)		

**Table 2:** Selected bond lengths (Å) for **2** [Chem scheme1]

Ni1—N1	2.0528 (16)	Ni1—N21	2.1224 (13)
Ni1—N11	2.1235 (14)		

**Table 3:** Selected bond lengths (Å) for **3** [Chem scheme1]

Ni1—N1	2.0517 (11)	Ni1—N21	2.1266 (10)
Ni1—N2	2.0552 (11)	Ni1—N31	2.1523 (11)
Ni1—N11	2.1358 (10)	Ni1—N41	2.1291 (11)

**Table 4:** Hydrogen-bond geometry (Å, °) for **1** [Chem scheme1]

*D*—H⋯*A*	*D*—H	H⋯*A*	*D*⋯*A*	*D*—H⋯*A*
C15—H15⋯O31^i^	0.95	2.61	3.373 (2)	138
O31—H31⋯S1	0.88 (4)	2.46 (4)	3.3379 (17)	172 (2)

Symmetry code: (i) 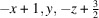 .

**Table 5:** Experimental details

	**1**	**2**	**3**
Crystal data			
Chemical formula	[Ni(NCS)_2_(C_6_H_7_N)_4_]·2C_2_H_6_O	[Ni(NCS)_2_(C_6_H_7_N)_4_]·2C_2_H_3_N	[Ni(NCS)_2_(C_6_H_7_N)_4_]·C_4_H_10_O
*M*_r_	639.51	629.48	621.49
Crystal system, space group	Monoclinic, *C*2/*c*	Monoclinic, *C*2/*c*	Monoclinic, *P*2_1_/*n*
Temperature (K)	100	100	100
*a*, *b*, *c* (Å)	18.5763 (1), 11.6179 (1), 15.8998 (1)	18.7990 (1), 11.3033 (1), 15.8639 (1)	10.2275 (10), 25.0468 (1), 12.7180 (1)
β (°)	97.448 (1)	96.825 (1)	94.600 (1)
*V* (Å^3^)	3402.51 (4)	3347.04 (4)	3247.4 (3)
*Z*	4	4	4
Radiation type	Cu *K*α	Cu *K*α	Cu *K*α
μ (mm^−1^)	2.24	2.25	2.31
Crystal size (mm)	0.2 × 0.1 × 0.05	0.25 × 0.15 × 0.05	0.2 × 0.2 × 0.15

Data collection			
Diffractometer	XtaLAB Synergy, Dualflex, HyPix	XtaLAB Synergy, Dualflex, HyPix	XtaLAB Synergy, Dualflex, HyPix
Absorption correction	Multi-scan (*CrysAlis PRO*; Rigaku OD, 2021 ▸)	Multi-scan (*CrysAlis PRO*; Rigaku OD, 2021 ▸)	Multi-scan (*CrysAlis PRO*; Rigaku OD, 2021 ▸)
*T*_min_, *T*_max_	0.857, 1.000	0.746, 1.000	0.933, 1.000
No. of measured, independent and observed [*I* > 2σ(*I*)] reflections	36997, 3672, 3589	35196, 3605, 3462	56224, 6974, 6907
*R*_int_	0.018	0.021	0.020
(sin θ/λ)_max_ (Å^−1^)	0.638	0.638	0.638

Refinement			
*R*[*F* ^2^ > 2σ(*F* ^2^)], *wR*(*F* ^2^), *S*	0.036, 0.098, 1.09	0.046, 0.153, 1.07	0.029, 0.076, 1.03
No. of reflections	3672	3605	6974
No. of parameters	213	264	368
No. of restraints	1	82	0
H-atom treatment	H atoms treated by a mixture of independent and constrained refinement	H-atom parameters constrained	H-atom parameters constrained
Δρ_max_, Δρ_min_ (e Å^−3^)	0.44, −0.38	0.80, −0.46	0.54, −0.32

Computer programs: *CrysAlis PRO* (Rigaku OD, 2021 ▸), *SHELXT2014/5* (Sheldrick, 2015*a* ▸), *SHELXL2016/6* (Sheldrick, 2015*b* ▸), *DIAMOND* (Brandenburg & Putz, 1999 ▸) and *publCIF* (Westrip, 2010 ▸).

## supplementary crystallographic information

### Bis(isothiocyanato-κN)tetrakis(3-methylpyridine-κN)\ nickel(II) ethanol disolvate (1) Crystal data

**Table:**

[Ni(NCS)_2_(C_6_H_7_N)_4_]·2C_2_H_6_O	*F*(000) = 1352
*M**_r_* = 639.51	*D*_x_ = 1.248 Mg m^−^^3^
Monoclinic, *C*2/*c*	Cu *K*α radiation, λ = 1.54184 Å
*a* = 18.5763 (1) Å	Cell parameters from 28027 reflections
*b* = 11.6179 (1) Å	θ = 4.5–79.4°
*c* = 15.8998 (1) Å	µ = 2.24 mm^−^^1^
β = 97.448 (1)°	*T* = 100 K
*V* = 3402.51 (4) Å^3^	Block, light purple
*Z* = 4	0.2 × 0.1 × 0.05 mm

### Bis(isothiocyanato-κN)tetrakis(3-methylpyridine-κN)\ nickel(II) ethanol disolvate (1) Data collection

**Table:**

XtaLAB Synergy, Dualflex, HyPix diffractometer	3672 independent reflections
Radiation source: micro-focus sealed X-ray tube, PhotonJet (Cu) X-ray Source	3589 reflections with *I* > 2σ(*I*)
Mirror monochromator	*R*_int_ = 0.018
Detector resolution: 10.0000 pixels mm^-1^	θ_max_ = 79.7°, θ_min_ = 4.5°
ω scans	*h* = −23→21
Absorption correction: multi-scan (CrysAlisPro; Rigaku OD, 2021)	*k* = −14→14
*T*_min_ = 0.857, *T*_max_ = 1.000	*l* = −19→20
36997 measured reflections	

### Bis(isothiocyanato-κN)tetrakis(3-methylpyridine-κN)\ nickel(II) ethanol disolvate (1) Refinement

**Table:**

Refinement on *F*^2^	Hydrogen site location: mixed
Least-squares matrix: full	H atoms treated by a mixture of independent and constrained refinement
*R*[*F*^2^ > 2σ(*F*^2^)] = 0.036	*w* = 1/[σ^2^(*F*_o_^2^) + (0.0454*P*)^2^ + 3.9194*P*] where *P* = (*F*_o_^2^ + 2*F*_c_^2^)/3
*wR*(*F*^2^) = 0.098	(Δ/σ)_max_ = 0.001
*S* = 1.09	Δρ_max_ = 0.44 e Å^−^^3^
3672 reflections	Δρ_min_ = −0.38 e Å^−^^3^
213 parameters	Extinction correction: SHELXL-2016/6 (Sheldrick 2015b), Fc^*^=kFc[1+0.001xFc^2^λ^3^/sin(2θ)]^-1/4^
1 restraint	Extinction coefficient: 0.00015 (4)
Primary atom site location: dual	

### Bis(isothiocyanato-κN)tetrakis(3-methylpyridine-κN)\ nickel(II) ethanol disolvate (1) Special details

**Table:**

Geometry. All esds (except the esd in the dihedral angle between two l.s. planes) are estimated using the full covariance matrix. The cell esds are taken into account individually in the estimation of esds in distances, angles and torsion angles; correlations between esds in cell parameters are only used when they are defined by crystal symmetry. An approximate (isotropic) treatment of cell esds is used for estimating esds involving l.s. planes.

### Bis(isothiocyanato-κN)tetrakis(3-methylpyridine-κN)\ nickel(II) ethanol disolvate (1) Fractional atomic coordinates and isotropic or equivalent isotropic displacement parameters (Å^2^)

**Table:**

	*x*	*y*	*z*	*U*_iso_*/*U*_eq_	Occ. (<1)
Ni1	0.500000	0.25420 (2)	0.750000	0.01790 (12)	
N1	0.42011 (7)	0.25422 (10)	0.82854 (8)	0.0245 (3)	
C1	0.36585 (8)	0.25217 (11)	0.85683 (9)	0.0221 (3)	
S1	0.28878 (2)	0.24794 (3)	0.89699 (2)	0.03098 (12)	
N11	0.55731 (6)	0.38324 (10)	0.82555 (7)	0.0245 (2)	
C11	0.52238 (9)	0.47165 (12)	0.85584 (9)	0.0288 (3)	
H11	0.470896	0.473317	0.844620	0.035*	
C12	0.55721 (10)	0.56143 (14)	0.90293 (11)	0.0398 (4)	
C13	0.63193 (12)	0.5568 (2)	0.91929 (16)	0.0632 (7)	
H13	0.657909	0.615808	0.951651	0.076*	
C14	0.66863 (11)	0.4666 (2)	0.88857 (16)	0.0678 (7)	
H14	0.720101	0.462974	0.899044	0.081*	
C15	0.62980 (9)	0.38109 (16)	0.84226 (11)	0.0393 (4)	
H15	0.655461	0.318635	0.821579	0.047*	
C16	0.51427 (13)	0.65865 (17)	0.93377 (14)	0.0536 (5)	
H16A	0.506739	0.718348	0.889971	0.080*	
H16B	0.540910	0.691251	0.985543	0.080*	
H16C	0.467130	0.629800	0.945930	0.080*	
N21	0.44290 (6)	0.12465 (10)	0.67471 (7)	0.0212 (2)	
C21	0.43798 (7)	0.12692 (12)	0.58975 (8)	0.0220 (3)	
H21	0.460160	0.189164	0.564102	0.026*	
C22	0.40237 (7)	0.04368 (12)	0.53708 (8)	0.0248 (3)	
C23	0.37036 (9)	−0.04668 (14)	0.57566 (10)	0.0329 (3)	
H23	0.345507	−0.105716	0.542233	0.039*	
C24	0.37492 (10)	−0.05012 (15)	0.66333 (10)	0.0381 (4)	
H24	0.353266	−0.111351	0.690695	0.046*	
C25	0.41140 (8)	0.03678 (13)	0.71022 (9)	0.0294 (3)	
H25	0.414269	0.034090	0.770276	0.035*	
C26	0.39962 (9)	0.05215 (14)	0.44214 (9)	0.0323 (3)	
H26A	0.361753	0.106922	0.419902	0.048*	
H26B	0.388635	−0.023684	0.416676	0.048*	
H26C	0.446699	0.078720	0.428104	0.048*	
O31	0.21100 (8)	0.26594 (14)	0.69605 (11)	0.0553 (4)	
H31	0.2297 (17)	0.253 (2)	0.749 (2)	0.081 (10)*	
C31	0.2139 (2)	0.1760 (4)	0.6416 (3)	0.0422 (8)	0.5
H31A	0.263961	0.165811	0.627965	0.051*	0.5
H31B	0.181366	0.190337	0.588223	0.051*	0.5
C32	0.1896 (3)	0.0696 (4)	0.6854 (4)	0.0599 (13)	0.5
H32A	0.224565	0.052494	0.735440	0.090*	0.5
H32B	0.187016	0.004215	0.646123	0.090*	0.5
H32C	0.141655	0.083178	0.702817	0.090*	0.5
C31'	0.2032 (3)	0.1313 (6)	0.6797 (3)	0.0653 (13)	0.5
H31C	0.244289	0.088968	0.711492	0.078*	0.5
H31D	0.157185	0.101977	0.696669	0.078*	0.5
C32'	0.2038 (3)	0.1196 (5)	0.5860 (3)	0.0780 (16)	0.5
H32D	0.162597	0.161779	0.555935	0.117*	0.5
H32E	0.200058	0.038053	0.570215	0.117*	0.5
H32F	0.249214	0.151212	0.570516	0.117*	0.5

### Bis(isothiocyanato-κN)tetrakis(3-methylpyridine-κN)\ nickel(II) ethanol disolvate (1) Atomic displacement parameters (Å^2^)

**Table:**

	*U*^11^	*U*^22^	*U*^33^	*U*^12^	*U*^13^	*U*^23^
Ni1	0.01946 (19)	0.01906 (18)	0.01550 (18)	0.000	0.00346 (12)	0.000
N1	0.0232 (6)	0.0273 (6)	0.0235 (6)	0.0014 (4)	0.0054 (5)	0.0020 (4)
C1	0.0271 (7)	0.0214 (6)	0.0178 (6)	0.0030 (5)	0.0024 (5)	0.0015 (4)
S1	0.0215 (2)	0.0433 (2)	0.0295 (2)	0.00391 (13)	0.00857 (15)	0.00623 (14)
N11	0.0283 (6)	0.0239 (6)	0.0218 (5)	−0.0037 (5)	0.0046 (4)	−0.0031 (4)
C11	0.0368 (8)	0.0249 (7)	0.0257 (7)	−0.0014 (6)	0.0079 (6)	−0.0026 (5)
C12	0.0528 (10)	0.0317 (8)	0.0383 (8)	−0.0107 (7)	0.0190 (7)	−0.0116 (7)
C13	0.0501 (11)	0.0683 (14)	0.0754 (15)	−0.0315 (10)	0.0244 (10)	−0.0458 (12)
C14	0.0319 (9)	0.0880 (17)	0.0852 (16)	−0.0214 (10)	0.0138 (10)	−0.0533 (14)
C15	0.0272 (7)	0.0477 (10)	0.0435 (9)	−0.0073 (7)	0.0067 (7)	−0.0188 (8)
C16	0.0740 (14)	0.0356 (9)	0.0547 (11)	−0.0057 (9)	0.0221 (10)	−0.0198 (8)
N21	0.0231 (5)	0.0233 (5)	0.0172 (5)	−0.0032 (4)	0.0031 (4)	−0.0004 (4)
C21	0.0230 (6)	0.0247 (6)	0.0184 (6)	−0.0016 (5)	0.0034 (5)	0.0015 (5)
C22	0.0242 (6)	0.0297 (7)	0.0202 (6)	−0.0010 (5)	0.0019 (5)	−0.0022 (5)
C23	0.0371 (8)	0.0324 (8)	0.0290 (7)	−0.0132 (6)	0.0034 (6)	−0.0058 (6)
C24	0.0504 (10)	0.0354 (8)	0.0295 (8)	−0.0206 (7)	0.0091 (7)	0.0001 (6)
C25	0.0379 (8)	0.0315 (7)	0.0195 (6)	−0.0102 (6)	0.0057 (6)	0.0019 (5)
C26	0.0393 (8)	0.0371 (8)	0.0197 (7)	−0.0045 (6)	0.0014 (6)	−0.0043 (6)
O31	0.0430 (8)	0.0768 (11)	0.0444 (8)	0.0050 (7)	−0.0006 (6)	−0.0149 (7)
C31	0.0380 (19)	0.048 (2)	0.041 (2)	−0.0017 (16)	0.0067 (16)	−0.0083 (19)
C32	0.052 (3)	0.041 (2)	0.089 (4)	−0.016 (2)	0.018 (2)	−0.013 (3)
C31'	0.075 (4)	0.068 (4)	0.050 (3)	−0.007 (3)	−0.006 (3)	0.001 (3)
C32'	0.093 (4)	0.077 (3)	0.058 (3)	0.021 (3)	−0.010 (3)	−0.020 (3)

### Bis(isothiocyanato-κN)tetrakis(3-methylpyridine-κN)\ nickel(II) ethanol disolvate (1) Geometric parameters (Å, º)

**Table:**

Ni1—N1^i^	2.0596 (13)	C22—C23	1.388 (2)
Ni1—N1	2.0597 (13)	C22—C26	1.5070 (19)
Ni1—N11^i^	2.1195 (12)	C23—H23	0.9500
Ni1—N11	2.1196 (12)	C23—C24	1.386 (2)
Ni1—N21	2.1200 (11)	C24—H24	0.9500
Ni1—N21^i^	2.1200 (11)	C24—C25	1.380 (2)
N1—C1	1.156 (2)	C25—H25	0.9500
C1—S1	1.6423 (15)	C26—H26A	0.9800
N11—C11	1.3375 (19)	C26—H26B	0.9800
N11—C15	1.339 (2)	C26—H26C	0.9800
C11—H11	0.9500	O31—H31	0.88 (4)
C11—C12	1.393 (2)	O31—C31	1.362 (4)
C12—C13	1.380 (3)	O31—C31'	1.589 (7)
C12—C16	1.501 (2)	C31—H31A	0.9900
C13—H13	0.9500	C31—H31B	0.9900
C13—C14	1.375 (3)	C31—C32	1.515 (6)
C14—H14	0.9500	C32—H32A	0.9800
C14—C15	1.382 (2)	C32—H32B	0.9800
C15—H15	0.9500	C32—H32C	0.9800
C16—H16A	0.9800	C31'—H31C	0.9900
C16—H16B	0.9800	C31'—H31D	0.9900
C16—H16C	0.9800	C31'—C32'	1.498 (7)
N21—C21	1.3423 (16)	C32'—H32D	0.9800
N21—C25	1.3376 (17)	C32'—H32E	0.9800
C21—H21	0.9500	C32'—H32F	0.9800
C21—C22	1.3894 (19)		
			
N1^i^—Ni1—N1	179.98 (6)	C22—C21—H21	118.0
N1—Ni1—N11	90.27 (5)	C21—C22—C26	120.66 (13)
N1^i^—Ni1—N11	89.72 (5)	C23—C22—C21	117.25 (12)
N1—Ni1—N11^i^	89.72 (5)	C23—C22—C26	122.09 (13)
N1^i^—Ni1—N11^i^	90.27 (5)	C22—C23—H23	120.2
N1—Ni1—N21^i^	90.27 (4)	C24—C23—C22	119.50 (14)
N1^i^—Ni1—N21^i^	89.74 (5)	C24—C23—H23	120.2
N1^i^—Ni1—N21	90.27 (4)	C23—C24—H24	120.5
N1—Ni1—N21	89.74 (5)	C25—C24—C23	118.95 (14)
N11^i^—Ni1—N11	89.96 (7)	C25—C24—H24	120.5
N11^i^—Ni1—N21	90.25 (5)	N21—C25—C24	122.79 (13)
N11—Ni1—N21^i^	90.25 (5)	N21—C25—H25	118.6
N11—Ni1—N21	179.79 (5)	C24—C25—H25	118.6
N11^i^—Ni1—N21^i^	179.79 (5)	C22—C26—H26A	109.5
N21^i^—Ni1—N21	89.53 (6)	C22—C26—H26B	109.5
C1—N1—Ni1	165.69 (12)	C22—C26—H26C	109.5
N1—C1—S1	179.47 (12)	H26A—C26—H26B	109.5
C11—N11—Ni1	121.04 (10)	H26A—C26—H26C	109.5
C11—N11—C15	117.74 (13)	H26B—C26—H26C	109.5
C15—N11—Ni1	121.18 (10)	C31—O31—H31	116.0 (18)
N11—C11—H11	118.1	C31'—O31—H31	90.7 (18)
N11—C11—C12	123.72 (15)	O31—C31—H31A	110.3
C12—C11—H11	118.1	O31—C31—H31B	110.3
C11—C12—C16	120.62 (17)	O31—C31—C32	107.2 (4)
C13—C12—C11	117.27 (16)	H31A—C31—H31B	108.5
C13—C12—C16	122.11 (17)	C32—C31—H31A	110.3
C12—C13—H13	120.1	C32—C31—H31B	110.3
C14—C13—C12	119.71 (17)	C31—C32—H32A	109.5
C14—C13—H13	120.1	C31—C32—H32B	109.5
C13—C14—H14	120.4	C31—C32—H32C	109.5
C13—C14—C15	119.27 (18)	H32A—C32—H32B	109.5
C15—C14—H14	120.4	H32A—C32—H32C	109.5
N11—C15—C14	122.30 (17)	H32B—C32—H32C	109.5
N11—C15—H15	118.9	O31—C31'—H31C	111.0
C14—C15—H15	118.9	O31—C31'—H31D	111.0
C12—C16—H16A	109.5	H31C—C31'—H31D	109.0
C12—C16—H16B	109.5	C32'—C31'—O31	103.9 (5)
C12—C16—H16C	109.5	C32'—C31'—H31C	111.0
H16A—C16—H16B	109.5	C32'—C31'—H31D	111.0
H16A—C16—H16C	109.5	C31'—C32'—H32D	109.5
H16B—C16—H16C	109.5	C31'—C32'—H32E	109.5
C21—N21—Ni1	121.21 (9)	C31'—C32'—H32F	109.5
C25—N21—Ni1	121.18 (9)	H32D—C32'—H32E	109.5
C25—N21—C21	117.61 (12)	H32D—C32'—H32F	109.5
N21—C21—H21	118.0	H32E—C32'—H32F	109.5
N21—C21—C22	123.90 (12)		

Symmetry code: (i) −*x*+1, *y*, −*z*+3/2.

### Bis(isothiocyanato-κN)tetrakis(3-methylpyridine-κN)\ nickel(II) ethanol disolvate (1) Hydrogen-bond geometry (Å, º)

**Table:**

*D*—H···*A*	*D*—H	H···*A*	*D*···*A*	*D*—H···*A*
C15—H15···O31^i^	0.95	2.61	3.373 (2)	138
O31—H31···S1	0.88 (4)	2.46 (4)	3.3379 (17)	172 (2)

Symmetry code: (i) −*x*+1, *y*, −*z*+3/2.

### Bis(isothiocyanato-κN)tetrakis(3-methylpyridine-κN)\ nickel(II) acetonitrile disolvate (2) Crystal data

**Table:**

[Ni(NCS)_2_(C_6_H_7_N)_4_]·2C_2_H_3_N	*F*(000) = 1320
*M**_r_* = 629.48	*D*_x_ = 1.249 Mg m^−^^3^
Monoclinic, *C*2/*c*	Cu *K*α radiation, λ = 1.54184 Å
*a* = 18.7990 (1) Å	Cell parameters from 26840 reflections
*b* = 11.3033 (1) Å	θ = 4.5–79.2°
*c* = 15.8639 (1) Å	µ = 2.25 mm^−^^1^
β = 96.825 (1)°	*T* = 100 K
*V* = 3347.04 (4) Å^3^	Block, light purple
*Z* = 4	0.25 × 0.15 × 0.05 mm

### Bis(isothiocyanato-κN)tetrakis(3-methylpyridine-κN)\ nickel(II) acetonitrile disolvate (2) Data collection

**Table:**

XtaLAB Synergy, Dualflex, HyPix diffractometer	3605 independent reflections
Radiation source: micro-focus sealed X-ray tube, PhotonJet (Cu) X-ray Source	3462 reflections with *I* > 2σ(*I*)
Mirror monochromator	*R*_int_ = 0.021
Detector resolution: 10.0000 pixels mm^-1^	θ_max_ = 79.7°, θ_min_ = 4.6°
ω scans	*h* = −23→21
Absorption correction: multi-scan (CrysAlisPro; Rigaku OD, 2021)	*k* = −13→14
*T*_min_ = 0.746, *T*_max_ = 1.000	*l* = −20→19
35196 measured reflections	

### Bis(isothiocyanato-κN)tetrakis(3-methylpyridine-κN)\ nickel(II) acetonitrile disolvate (2) Refinement

**Table:**

Refinement on *F*^2^	Primary atom site location: dual
Least-squares matrix: full	Hydrogen site location: inferred from neighbouring sites
*R*[*F*^2^ > 2σ(*F*^2^)] = 0.046	H-atom parameters constrained
*wR*(*F*^2^) = 0.153	*w* = 1/[σ^2^(*F*_o_^2^) + (0.0965*P*)^2^ + 2.692*P*] where *P* = (*F*_o_^2^ + 2*F*_c_^2^)/3
*S* = 1.07	(Δ/σ)_max_ = 0.001
3605 reflections	Δρ_max_ = 0.80 e Å^−^^3^
264 parameters	Δρ_min_ = −0.46 e Å^−^^3^
82 restraints	

### Bis(isothiocyanato-κN)tetrakis(3-methylpyridine-κN)\ nickel(II) acetonitrile disolvate (2) Special details

**Table:**

Geometry. All esds (except the esd in the dihedral angle between two l.s. planes) are estimated using the full covariance matrix. The cell esds are taken into account individually in the estimation of esds in distances, angles and torsion angles; correlations between esds in cell parameters are only used when they are defined by crystal symmetry. An approximate (isotropic) treatment of cell esds is used for estimating esds involving l.s. planes.

### Bis(isothiocyanato-κN)tetrakis(3-methylpyridine-κN)\ nickel(II) acetonitrile disolvate (2) Fractional atomic coordinates and isotropic or equivalent isotropic displacement parameters (Å^2^)

**Table:**

	*x*	*y*	*z*	*U*_iso_*/*U*_eq_	Occ. (<1)
Ni1	0.500000	0.24890 (3)	0.750000	0.02595 (17)	
N1	0.42021 (9)	0.24892 (10)	0.82781 (11)	0.0345 (4)	
C1	0.36511 (10)	0.24794 (11)	0.85346 (11)	0.0284 (4)	
S1	0.28727 (2)	0.24611 (4)	0.88985 (3)	0.04010 (18)	
N11	0.55665 (7)	0.38053 (12)	0.82637 (9)	0.0330 (3)	
C11	0.52259 (10)	0.47154 (15)	0.85750 (11)	0.0386 (4)	
H11	0.472097	0.476613	0.843833	0.046*	
C12	0.55697 (12)	0.55942 (17)	0.90889 (13)	0.0503 (5)	
C13	0.63026 (13)	0.5497 (2)	0.92937 (16)	0.0608 (6)	
H13	0.655637	0.607165	0.964830	0.073*	
C14	0.66640 (11)	0.4564 (2)	0.89825 (16)	0.0612 (6)	
H14	0.716769	0.448707	0.911946	0.073*	
C15	0.62794 (9)	0.37415 (17)	0.84660 (12)	0.0449 (4)	
H15	0.653079	0.310505	0.824553	0.054*	
C16	0.51514 (16)	0.6613 (2)	0.93882 (19)	0.0760 (8)	
H16A	0.509929	0.722969	0.895090	0.114*	
H16B	0.540591	0.693673	0.991274	0.114*	
H16C	0.467636	0.633742	0.949620	0.114*	
N21	0.44432 (7)	0.11538 (12)	0.67464 (8)	0.0310 (3)	
C21	0.43764 (9)	0.12022 (14)	0.58979 (10)	0.0337 (3)	
H21	0.460555	0.183760	0.564569	0.040*	0.757 (5)
H21A	0.452665	0.188327	0.561597	0.040*	0.243 (5)
C22	0.4005 (3)	0.0410 (5)	0.5368 (4)	0.0347 (12)	0.757 (5)
C23	0.3683 (3)	−0.0535 (4)	0.5741 (3)	0.0400 (9)	0.757 (5)
H23	0.343460	−0.112681	0.539724	0.048*	0.757 (5)
C24	0.3730 (2)	−0.0603 (3)	0.6615 (2)	0.0453 (8)	0.757 (5)
H24	0.350897	−0.123617	0.687939	0.054*	0.757 (5)
C25	0.4100 (2)	0.0254 (5)	0.7095 (4)	0.0380 (11)	0.757 (5)
H25	0.411687	0.021686	0.769528	0.046*	0.757 (5)
C26	0.39812 (15)	0.0533 (2)	0.44197 (14)	0.0466 (7)	0.757 (5)
H26A	0.368378	0.121456	0.422608	0.070*	0.757 (5)
H26B	0.377644	−0.018657	0.414374	0.070*	0.757 (5)
H26C	0.446794	0.064930	0.427212	0.070*	0.757 (5)
C22'	0.4062 (13)	0.017 (2)	0.5403 (11)	0.042 (4)	0.243 (5)
H22'	0.398402	0.020171	0.480103	0.051*	0.243 (5)
C23'	0.3890 (8)	−0.0810 (15)	0.5813 (11)	0.048 (3)	0.243 (5)
H23'	0.367339	−0.146067	0.550209	0.058*	0.243 (5)
C24'	0.4025 (5)	−0.0869 (9)	0.6675 (6)	0.0368 (19)	0.243 (5)
C25'	0.4322 (6)	0.0147 (15)	0.7115 (12)	0.027 (2)	0.243 (5)
H25'	0.444086	0.009232	0.771277	0.033*	0.243 (5)
C26'	0.3910 (5)	−0.1978 (8)	0.7179 (5)	0.054 (2)	0.243 (5)
H26D	0.349846	−0.241696	0.689851	0.081*	0.243 (5)
H26E	0.381743	−0.175834	0.775373	0.081*	0.243 (5)
H26F	0.433910	−0.247524	0.721077	0.081*	0.243 (5)
N31	0.3955 (6)	0.6917 (11)	0.7911 (7)	0.164 (3)	0.5
C31	0.3454 (6)	0.6475 (8)	0.8069 (6)	0.126 (2)	0.5
C32	0.2775 (8)	0.5919 (12)	0.8173 (13)	0.160 (5)	0.5
H32A	0.241992	0.613118	0.769401	0.239*	0.5
H32B	0.261097	0.619130	0.870340	0.239*	0.5
H32C	0.283598	0.505767	0.819183	0.239*	0.5
N31'	0.2788 (5)	0.7116 (10)	0.9198 (8)	0.163 (3)	0.5
C31'	0.2854 (4)	0.6431 (8)	0.8677 (8)	0.113 (2)	0.5
C32'	0.2937 (5)	0.5588 (9)	0.8067 (11)	0.122 (3)	0.5
H32D	0.328828	0.586604	0.770166	0.182*	0.5
H32E	0.247670	0.545062	0.772217	0.182*	0.5
H32F	0.310663	0.484778	0.834360	0.182*	0.5

### Bis(isothiocyanato-κN)tetrakis(3-methylpyridine-κN)\ nickel(II) acetonitrile disolvate (2) Atomic displacement parameters (Å^2^)

**Table:**

	*U*^11^	*U*^22^	*U*^33^	*U*^12^	*U*^13^	*U*^23^
Ni1	0.0266 (3)	0.0241 (3)	0.0272 (3)	0.000	0.00339 (17)	0.000
N1	0.0328 (8)	0.0347 (8)	0.0367 (8)	−0.0004 (4)	0.0076 (6)	0.0012 (5)
C1	0.0336 (8)	0.0257 (8)	0.0254 (8)	0.0016 (5)	0.0009 (6)	0.0006 (4)
S1	0.0297 (3)	0.0488 (3)	0.0431 (3)	0.00236 (14)	0.0093 (2)	0.00200 (15)
N11	0.0360 (7)	0.0286 (6)	0.0346 (7)	−0.0035 (5)	0.0044 (5)	−0.0037 (5)
C11	0.0452 (9)	0.0324 (8)	0.0395 (8)	−0.0026 (7)	0.0099 (7)	−0.0057 (6)
C12	0.0649 (12)	0.0391 (9)	0.0491 (10)	−0.0099 (8)	0.0166 (9)	−0.0137 (8)
C13	0.0609 (13)	0.0601 (13)	0.0611 (13)	−0.0223 (11)	0.0067 (10)	−0.0259 (11)
C14	0.0425 (10)	0.0660 (14)	0.0729 (14)	−0.0132 (9)	−0.0023 (9)	−0.0211 (11)
C15	0.0364 (8)	0.0431 (9)	0.0538 (10)	−0.0042 (7)	0.0001 (7)	−0.0112 (8)
C16	0.0889 (19)	0.0580 (14)	0.0850 (18)	−0.0064 (12)	0.0269 (15)	−0.0332 (13)
N21	0.0352 (7)	0.0295 (6)	0.0276 (6)	−0.0048 (5)	0.0008 (5)	0.0009 (5)
C21	0.0393 (8)	0.0330 (8)	0.0288 (7)	−0.0021 (6)	0.0038 (6)	0.0018 (6)
C22	0.0409 (18)	0.031 (2)	0.0324 (18)	−0.0011 (17)	0.0058 (14)	0.0012 (12)
C23	0.046 (2)	0.037 (2)	0.0369 (16)	−0.0138 (16)	0.0011 (16)	−0.0052 (13)
C24	0.054 (2)	0.0409 (17)	0.0411 (14)	−0.0197 (15)	0.0044 (15)	0.0019 (12)
C25	0.041 (3)	0.0412 (18)	0.0315 (15)	−0.014 (2)	0.001 (2)	0.0053 (12)
C26	0.0616 (15)	0.0482 (14)	0.0294 (11)	−0.0122 (11)	0.0032 (10)	−0.0058 (9)
C22'	0.064 (8)	0.037 (7)	0.019 (4)	0.014 (5)	−0.020 (4)	−0.012 (4)
C23'	0.053 (8)	0.042 (6)	0.045 (4)	0.000 (5)	−0.012 (5)	−0.009 (4)
C24'	0.034 (5)	0.036 (4)	0.038 (3)	−0.004 (3)	−0.004 (3)	−0.004 (3)
C25'	0.021 (6)	0.030 (4)	0.029 (4)	−0.006 (4)	−0.008 (4)	−0.002 (3)
C26'	0.063 (5)	0.045 (4)	0.050 (4)	−0.025 (4)	−0.010 (4)	0.001 (3)
N31	0.188 (4)	0.153 (7)	0.146 (7)	0.007 (4)	0.003 (4)	0.067 (6)
C31	0.178 (4)	0.098 (5)	0.096 (5)	0.025 (3)	−0.002 (4)	0.040 (4)
C32	0.172 (5)	0.099 (9)	0.202 (14)	0.038 (4)	0.000 (5)	0.092 (9)
N31'	0.128 (7)	0.131 (5)	0.221 (5)	0.049 (5)	−0.016 (4)	0.031 (4)
C31'	0.051 (3)	0.087 (4)	0.197 (5)	0.011 (3)	−0.001 (4)	0.062 (3)
C32'	0.100 (5)	0.077 (4)	0.184 (5)	0.025 (4)	0.000 (4)	0.076 (3)

### Bis(isothiocyanato-κN)tetrakis(3-methylpyridine-κN)\ nickel(II) acetonitrile disolvate (2) Geometric parameters (Å, º)

**Table:**

Ni1—N1^i^	2.0528 (16)	C22—C26	1.506 (6)
Ni1—N1	2.0528 (16)	C23—H23	0.9500
Ni1—N11	2.1235 (14)	C23—C24	1.381 (5)
Ni1—N11^i^	2.1235 (13)	C24—H24	0.9500
Ni1—N21	2.1224 (13)	C24—C25	1.370 (7)
Ni1—N21^i^	2.1224 (13)	C25—H25	0.9500
N1—C1	1.157 (3)	C26—H26A	0.9800
C1—S1	1.6358 (19)	C26—H26B	0.9800
N11—C11	1.337 (2)	C26—H26C	0.9800
N11—C15	1.342 (2)	C22'—H22'	0.9500
C11—H11	0.9500	C22'—C23'	1.34 (2)
C11—C12	1.394 (2)	C23'—H23'	0.9500
C12—C13	1.382 (3)	C23'—C24'	1.363 (19)
C12—C16	1.503 (3)	C24'—C25'	1.423 (19)
C13—H13	0.9500	C24'—C26'	1.516 (13)
C13—C14	1.377 (3)	C25'—H25'	0.9500
C14—H14	0.9500	C26'—H26D	0.9800
C14—C15	1.385 (3)	C26'—H26E	0.9800
C15—H15	0.9500	C26'—H26F	0.9800
C16—H16A	0.9800	N31—C31	1.121 (11)
C16—H16B	0.9800	C31—C32	1.449 (14)
C16—H16C	0.9800	C32—H32A	0.9800
N21—C21	1.3381 (19)	C32—H32B	0.9800
N21—C25	1.357 (6)	C32—H32C	0.9800
N21—C25'	1.312 (19)	N31'—C31'	1.151 (12)
C21—H21	0.9500	C31'—C32'	1.381 (15)
C21—H21A	0.9500	C32'—H32D	0.9800
C21—C22	1.363 (6)	C32'—H32E	0.9800
C21—C22'	1.490 (19)	C32'—H32F	0.9800
C22—C23	1.394 (6)		
			
N1^i^—Ni1—N1	179.99 (7)	C22'—C21—H21A	120.6
N1^i^—Ni1—N11^i^	90.55 (6)	C21—C22—C23	117.3 (4)
N1—Ni1—N11^i^	89.44 (6)	C21—C22—C26	120.5 (3)
N1—Ni1—N11	90.55 (6)	C23—C22—C26	122.1 (5)
N1^i^—Ni1—N11	89.44 (6)	C22—C23—H23	120.3
N1^i^—Ni1—N21	90.46 (6)	C24—C23—C22	119.3 (4)
N1—Ni1—N21	89.55 (6)	C24—C23—H23	120.3
N1—Ni1—N21^i^	90.46 (6)	C23—C24—H24	120.4
N1^i^—Ni1—N21^i^	89.55 (6)	C25—C24—C23	119.1 (4)
N11—Ni1—N11^i^	91.03 (8)	C25—C24—H24	120.4
N21^i^—Ni1—N11	89.80 (6)	N21—C25—C24	122.5 (5)
N21^i^—Ni1—N11^i^	179.16 (5)	N21—C25—H25	118.7
N21—Ni1—N11^i^	89.80 (6)	C24—C25—H25	118.7
N21—Ni1—N11	179.16 (5)	C22—C26—H26A	109.5
N21—Ni1—N21^i^	89.36 (7)	C22—C26—H26B	109.5
C1—N1—Ni1	163.77 (15)	C22—C26—H26C	109.5
N1—C1—S1	179.81 (16)	H26A—C26—H26B	109.5
C11—N11—Ni1	121.35 (11)	H26A—C26—H26C	109.5
C11—N11—C15	117.48 (15)	H26B—C26—H26C	109.5
C15—N11—Ni1	121.16 (11)	C21—C22'—H22'	120.2
N11—C11—H11	118.1	C23'—C22'—C21	119.6 (14)
N11—C11—C12	123.73 (17)	C23'—C22'—H22'	120.2
C12—C11—H11	118.1	C22'—C23'—H23'	120.0
C11—C12—C16	120.5 (2)	C22'—C23'—C24'	120.0 (15)
C13—C12—C11	117.42 (18)	C24'—C23'—H23'	120.0
C13—C12—C16	122.1 (2)	C23'—C24'—C25'	117.9 (13)
C12—C13—H13	120.1	C23'—C24'—C26'	123.3 (10)
C14—C13—C12	119.83 (19)	C25'—C24'—C26'	118.7 (11)
C14—C13—H13	120.1	N21—C25'—C24'	124.1 (15)
C13—C14—H14	120.6	N21—C25'—H25'	118.0
C13—C14—C15	118.7 (2)	C24'—C25'—H25'	118.0
C15—C14—H14	120.6	C24'—C26'—H26D	109.5
N11—C15—C14	122.78 (18)	C24'—C26'—H26E	109.5
N11—C15—H15	118.6	C24'—C26'—H26F	109.5
C14—C15—H15	118.6	H26D—C26'—H26E	109.5
C12—C16—H16A	109.5	H26D—C26'—H26F	109.5
C12—C16—H16B	109.5	H26E—C26'—H26F	109.5
C12—C16—H16C	109.5	N31—C31—C32	173.6 (12)
H16A—C16—H16B	109.5	C31—C32—H32A	109.5
H16A—C16—H16C	109.5	C31—C32—H32B	109.5
H16B—C16—H16C	109.5	C31—C32—H32C	109.5
C21—N21—Ni1	121.24 (10)	H32A—C32—H32B	109.5
C21—N21—C25	116.6 (3)	H32A—C32—H32C	109.5
C25—N21—Ni1	122.0 (3)	H32B—C32—H32C	109.5
C25'—N21—Ni1	117.8 (8)	N31'—C31'—C32'	178.5 (12)
C25'—N21—C21	118.9 (8)	C31'—C32'—H32D	109.5
N21—C21—H21	117.5	C31'—C32'—H32E	109.5
N21—C21—H21A	120.6	C31'—C32'—H32F	109.5
N21—C21—C22	125.0 (2)	H32D—C32'—H32E	109.5
N21—C21—C22'	118.8 (8)	H32D—C32'—H32F	109.5
C22—C21—H21	117.5	H32E—C32'—H32F	109.5

Symmetry code: (i) −*x*+1, *y*, −*z*+3/2.

### Bis(isothiocyanato-κN)tetrakis(3-methylpyridine-κN)\ nickel(II) acetonitrile disolvate (2) Hydrogen-bond geometry (Å, º)

**Table:**

*D*—H···*A*	*D*—H	H···*A*	*D*···*A*	*D*—H···*A*
C25′—H25′···N21^i^	0.95	2.48	2.991 (15)	114
C32—H32*A*···S1^ii^	0.98	2.93	3.791 (17)	147
C32′—H32*F*···S1	0.98	2.89	3.779 (10)	152

Symmetry codes: (i) −*x*+1, *y*, −*z*+3/2; (ii) −*x*+1/2, *y*+1/2, −*z*+3/2.

### Bis(isothiocyanato-κN)tetrakis(3-methylpyridine-κN)\ nickel(II) diethyl ether monosolvate (3) Crystal data

**Table:**

[Ni(NCS)_2_(C_6_H_7_N)_4_]·C_4_H_10_O	*F*(000) = 1312
*M**_r_* = 621.49	*D*_x_ = 1.271 Mg m^−^^3^
Monoclinic, *P*2_1_/*n*	Cu *K*α radiation, λ = 1.54184 Å
*a* = 10.2275 (10) Å	Cell parameters from 43692 reflections
*b* = 25.0468 (1) Å	θ = 3.5–79.0°
*c* = 12.7180 (1) Å	µ = 2.31 mm^−^^1^
β = 94.600 (1)°	*T* = 100 K
*V* = 3247.4 (3) Å^3^	Block, light purple
*Z* = 4	0.2 × 0.2 × 0.15 mm

### Bis(isothiocyanato-κN)tetrakis(3-methylpyridine-κN)\ nickel(II) diethyl ether monosolvate (3) Data collection

**Table:**

XtaLAB Synergy, Dualflex, HyPix diffractometer	6974 independent reflections
Radiation source: micro-focus sealed X-ray tube, PhotonJet (Cu) X-ray Source	6907 reflections with *I* > 2σ(*I*)
Mirror monochromator	*R*_int_ = 0.020
Detector resolution: 10.0000 pixels mm^-1^	θ_max_ = 79.6°, θ_min_ = 3.5°
ω scans	*h* = −12→13
Absorption correction: multi-scan (CrysAlisPro; Rigaku OD, 2021)	*k* = −31→30
*T*_min_ = 0.933, *T*_max_ = 1.000	*l* = −15→16
56224 measured reflections	

### Bis(isothiocyanato-κN)tetrakis(3-methylpyridine-κN)\ nickel(II) diethyl ether monosolvate (3) Refinement

**Table:**

Refinement on *F*^2^	Hydrogen site location: inferred from neighbouring sites
Least-squares matrix: full	H-atom parameters constrained
*R*[*F*^2^ > 2σ(*F*^2^)] = 0.029	*w* = 1/[σ^2^(*F*_o_^2^) + (0.0356*P*)^2^ + 1.9652*P*] where *P* = (*F*_o_^2^ + 2*F*_c_^2^)/3
*wR*(*F*^2^) = 0.076	(Δ/σ)_max_ = 0.002
*S* = 1.03	Δρ_max_ = 0.54 e Å^−^^3^
6974 reflections	Δρ_min_ = −0.32 e Å^−^^3^
368 parameters	Extinction correction: SHELXL-2016/6 (Sheldrick 2015b), Fc^*^=kFc[1+0.001xFc^2^λ^3^/sin(2θ)]^-1/4^
0 restraints	Extinction coefficient: 0.00020 (4)
Primary atom site location: dual	

### Bis(isothiocyanato-κN)tetrakis(3-methylpyridine-κN)\ nickel(II) diethyl ether monosolvate (3) Special details

**Table:**

Geometry. All esds (except the esd in the dihedral angle between two l.s. planes) are estimated using the full covariance matrix. The cell esds are taken into account individually in the estimation of esds in distances, angles and torsion angles; correlations between esds in cell parameters are only used when they are defined by crystal symmetry. An approximate (isotropic) treatment of cell esds is used for estimating esds involving l.s. planes.

### Bis(isothiocyanato-κN)tetrakis(3-methylpyridine-κN)\ nickel(II) diethyl ether monosolvate (3) Fractional atomic coordinates and isotropic or equivalent isotropic displacement parameters (Å^2^)

**Table:**

	*x*	*y*	*z*	*U*_iso_*/*U*_eq_	
Ni1	0.24076 (2)	0.61197 (2)	0.27926 (2)	0.01630 (7)	
N1	0.24400 (11)	0.60562 (4)	0.44028 (9)	0.0217 (2)	
C1	0.24082 (12)	0.62304 (5)	0.52491 (10)	0.0188 (2)	
S1	0.23850 (4)	0.64887 (2)	0.64199 (3)	0.03244 (9)	
N2	0.23270 (11)	0.61727 (4)	0.11760 (9)	0.0209 (2)	
C2	0.26680 (12)	0.62259 (5)	0.03386 (10)	0.0195 (2)	
S2	0.31719 (4)	0.63063 (2)	−0.08404 (3)	0.02805 (9)	
N11	0.11637 (10)	0.54346 (4)	0.26359 (8)	0.0182 (2)	
C11	0.12071 (12)	0.51046 (5)	0.18089 (10)	0.0195 (2)	
H11	0.180894	0.518406	0.129934	0.023*	
C12	0.04240 (12)	0.46529 (5)	0.16522 (10)	0.0212 (2)	
C13	−0.04471 (13)	0.45407 (5)	0.24070 (11)	0.0252 (3)	
H13	−0.100594	0.423786	0.233060	0.030*	
C14	−0.04931 (13)	0.48748 (6)	0.32726 (11)	0.0259 (3)	
H14	−0.107695	0.480129	0.379882	0.031*	
C15	0.03222 (12)	0.53170 (5)	0.33612 (10)	0.0218 (2)	
H15	0.028503	0.554484	0.395516	0.026*	
C16	0.05390 (14)	0.42992 (6)	0.07075 (11)	0.0286 (3)	
H16A	0.090667	0.450425	0.014493	0.043*	
H16B	−0.033150	0.416564	0.045699	0.043*	
H16C	0.111655	0.399722	0.090543	0.043*	
N21	0.40489 (10)	0.55986 (4)	0.27878 (8)	0.0181 (2)	
C21	0.48770 (12)	0.56126 (5)	0.20234 (10)	0.0209 (2)	
H21	0.479956	0.589974	0.153433	0.025*	
C22	0.58430 (13)	0.52316 (5)	0.19038 (11)	0.0249 (3)	
C23	0.59499 (13)	0.48144 (5)	0.26294 (11)	0.0251 (3)	
H23	0.658347	0.454105	0.256871	0.030*	
C24	0.51244 (13)	0.48016 (5)	0.34399 (11)	0.0240 (3)	
H24	0.519758	0.452469	0.395165	0.029*	
C25	0.41891 (12)	0.51993 (5)	0.34931 (10)	0.0208 (2)	
H25	0.362470	0.518900	0.405078	0.025*	
C26	0.67263 (16)	0.52747 (7)	0.10169 (14)	0.0393 (4)	
H26A	0.632509	0.551229	0.046997	0.059*	
H26B	0.685163	0.492004	0.071558	0.059*	
H26C	0.757741	0.541995	0.128735	0.059*	
N31	0.36520 (10)	0.68134 (4)	0.29236 (8)	0.0190 (2)	
C31	0.44241 (12)	0.69197 (5)	0.38051 (10)	0.0206 (2)	
H31	0.445853	0.666502	0.436028	0.025*	
C32	0.51760 (12)	0.73812 (5)	0.39515 (10)	0.0220 (3)	
C33	0.51474 (13)	0.77437 (5)	0.31195 (11)	0.0240 (3)	
H33	0.565253	0.806207	0.318239	0.029*	
C34	0.43767 (14)	0.76355 (5)	0.22017 (11)	0.0262 (3)	
H34	0.435711	0.787548	0.162214	0.031*	
C35	0.36329 (13)	0.71725 (5)	0.21377 (11)	0.0238 (3)	
H35	0.308631	0.710622	0.151170	0.029*	
C36	0.59863 (15)	0.74773 (6)	0.49744 (12)	0.0310 (3)	
H36A	0.567883	0.780167	0.530802	0.046*	
H36B	0.690902	0.752064	0.483540	0.046*	
H36C	0.589876	0.717217	0.544596	0.046*	
N41	0.07440 (10)	0.66297 (4)	0.28090 (9)	0.0202 (2)	
C41	−0.02476 (13)	0.65985 (5)	0.20552 (11)	0.0228 (3)	
H41	−0.020280	0.633303	0.152574	0.027*	
C42	−0.13370 (13)	0.69318 (6)	0.20032 (12)	0.0277 (3)	
C43	−0.13848 (14)	0.73168 (5)	0.27870 (12)	0.0291 (3)	
H43	−0.211005	0.755405	0.278184	0.035*	
C44	−0.03766 (15)	0.73536 (5)	0.35720 (12)	0.0291 (3)	
H44	−0.040057	0.761438	0.411257	0.035*	
C45	0.06728 (14)	0.70034 (5)	0.35589 (11)	0.0252 (3)	
H45	0.136618	0.702897	0.410014	0.030*	
C46	−0.24086 (16)	0.68799 (8)	0.11234 (16)	0.0458 (4)	
H46A	−0.215254	0.661426	0.061268	0.069*	
H46B	−0.254755	0.722555	0.077099	0.069*	
H46C	−0.322232	0.676613	0.141465	0.069*	
O1	0.69662 (10)	0.62578 (4)	0.59182 (9)	0.0317 (2)	
C51	0.73601 (16)	0.60727 (7)	0.41488 (14)	0.0366 (3)	
H51A	0.795731	0.589524	0.369494	0.055*	
H51B	0.732205	0.645494	0.398425	0.055*	
H51C	0.648145	0.591754	0.402625	0.055*	
C52	0.78480 (15)	0.59971 (6)	0.52804 (14)	0.0347 (3)	
H52A	0.789405	0.561178	0.545302	0.042*	
H52B	0.873782	0.615096	0.541081	0.042*	
C53	0.72920 (16)	0.61857 (6)	0.70157 (13)	0.0343 (3)	
H53A	0.820988	0.629759	0.720164	0.041*	
H53B	0.720701	0.580448	0.720396	0.041*	
C54	0.63737 (18)	0.65180 (8)	0.76094 (14)	0.0444 (4)	
H54A	0.546535	0.642006	0.738825	0.067*	
H54B	0.651052	0.689678	0.745921	0.067*	
H54C	0.654630	0.645388	0.836800	0.067*	

### Bis(isothiocyanato-κN)tetrakis(3-methylpyridine-κN)\ nickel(II) diethyl ether monosolvate (3) Atomic displacement parameters (Å^2^)

**Table:**

	*U*^11^	*U*^22^	*U*^33^	*U*^12^	*U*^13^	*U*^23^
Ni1	0.01686 (11)	0.01632 (11)	0.01561 (11)	0.00084 (7)	0.00063 (8)	−0.00079 (7)
N1	0.0249 (5)	0.0217 (5)	0.0185 (5)	0.0001 (4)	0.0018 (4)	−0.0013 (4)
C1	0.0193 (6)	0.0166 (5)	0.0206 (6)	−0.0011 (4)	0.0021 (4)	0.0029 (4)
S1	0.0513 (2)	0.02888 (18)	0.01775 (16)	0.00010 (15)	0.00635 (14)	−0.00430 (12)
N2	0.0198 (5)	0.0204 (5)	0.0223 (6)	0.0006 (4)	0.0008 (4)	0.0006 (4)
C2	0.0214 (6)	0.0145 (5)	0.0216 (6)	0.0021 (4)	−0.0046 (5)	−0.0029 (4)
S2	0.03759 (19)	0.02713 (17)	0.02051 (16)	−0.00213 (13)	0.00901 (13)	0.00014 (12)
N11	0.0171 (5)	0.0177 (5)	0.0196 (5)	0.0011 (4)	0.0002 (4)	0.0001 (4)
C11	0.0187 (5)	0.0204 (6)	0.0192 (6)	0.0007 (4)	0.0006 (4)	0.0002 (5)
C12	0.0197 (6)	0.0201 (6)	0.0232 (6)	0.0009 (5)	−0.0027 (5)	−0.0010 (5)
C13	0.0203 (6)	0.0228 (6)	0.0323 (7)	−0.0033 (5)	0.0007 (5)	−0.0002 (5)
C14	0.0209 (6)	0.0282 (7)	0.0294 (7)	−0.0023 (5)	0.0064 (5)	0.0006 (5)
C15	0.0189 (6)	0.0237 (6)	0.0229 (6)	0.0012 (5)	0.0029 (5)	−0.0014 (5)
C16	0.0313 (7)	0.0265 (7)	0.0276 (7)	−0.0049 (5)	0.0005 (5)	−0.0061 (5)
N21	0.0165 (5)	0.0175 (5)	0.0199 (5)	0.0002 (4)	−0.0003 (4)	−0.0010 (4)
C21	0.0190 (6)	0.0207 (6)	0.0230 (6)	0.0003 (5)	0.0015 (5)	0.0016 (5)
C22	0.0220 (6)	0.0244 (6)	0.0287 (7)	0.0018 (5)	0.0056 (5)	0.0001 (5)
C23	0.0218 (6)	0.0211 (6)	0.0326 (7)	0.0038 (5)	0.0024 (5)	−0.0010 (5)
C24	0.0247 (6)	0.0196 (6)	0.0275 (7)	0.0009 (5)	−0.0006 (5)	0.0031 (5)
C25	0.0205 (6)	0.0203 (6)	0.0215 (6)	−0.0003 (5)	0.0012 (5)	0.0010 (5)
C26	0.0373 (8)	0.0393 (9)	0.0441 (9)	0.0125 (7)	0.0206 (7)	0.0100 (7)
N31	0.0191 (5)	0.0164 (5)	0.0212 (5)	0.0008 (4)	0.0008 (4)	−0.0007 (4)
C31	0.0202 (6)	0.0200 (6)	0.0215 (6)	0.0005 (5)	0.0003 (5)	0.0002 (5)
C32	0.0206 (6)	0.0206 (6)	0.0245 (6)	0.0004 (5)	0.0001 (5)	−0.0030 (5)
C33	0.0240 (6)	0.0172 (6)	0.0310 (7)	−0.0012 (5)	0.0031 (5)	−0.0014 (5)
C34	0.0318 (7)	0.0193 (6)	0.0272 (7)	−0.0006 (5)	0.0002 (5)	0.0038 (5)
C35	0.0266 (6)	0.0203 (6)	0.0236 (6)	0.0007 (5)	−0.0026 (5)	0.0014 (5)
C36	0.0350 (7)	0.0260 (7)	0.0302 (7)	−0.0064 (6)	−0.0077 (6)	−0.0020 (6)
N41	0.0201 (5)	0.0185 (5)	0.0223 (5)	0.0022 (4)	0.0033 (4)	0.0007 (4)
C41	0.0216 (6)	0.0209 (6)	0.0259 (6)	0.0013 (5)	0.0021 (5)	0.0005 (5)
C42	0.0215 (6)	0.0251 (7)	0.0363 (8)	0.0030 (5)	0.0013 (5)	0.0052 (6)
C43	0.0257 (7)	0.0221 (6)	0.0407 (8)	0.0076 (5)	0.0092 (6)	0.0059 (6)
C44	0.0344 (7)	0.0207 (6)	0.0333 (7)	0.0062 (5)	0.0088 (6)	−0.0009 (5)
C45	0.0287 (7)	0.0208 (6)	0.0260 (7)	0.0036 (5)	0.0027 (5)	−0.0012 (5)
C46	0.0300 (8)	0.0435 (9)	0.0607 (11)	0.0116 (7)	−0.0155 (8)	−0.0055 (8)
O1	0.0290 (5)	0.0293 (5)	0.0369 (6)	0.0067 (4)	0.0030 (4)	0.0052 (4)
C51	0.0323 (8)	0.0337 (8)	0.0445 (9)	0.0039 (6)	0.0078 (7)	−0.0041 (7)
C52	0.0259 (7)	0.0278 (7)	0.0502 (9)	0.0042 (6)	0.0026 (6)	−0.0010 (7)
C53	0.0341 (8)	0.0298 (7)	0.0380 (8)	−0.0031 (6)	−0.0044 (6)	0.0070 (6)
C54	0.0454 (10)	0.0504 (10)	0.0375 (9)	−0.0008 (8)	0.0045 (7)	0.0028 (8)

### Bis(isothiocyanato-κN)tetrakis(3-methylpyridine-κN)\ nickel(II) diethyl ether monosolvate (3) Geometric parameters (Å, º)

**Table:**

Ni1—N1	2.0517 (11)	C31—H31	0.9500
Ni1—N2	2.0552 (11)	C31—C32	1.3926 (18)
Ni1—N11	2.1358 (10)	C32—C33	1.3928 (19)
Ni1—N21	2.1266 (10)	C32—C36	1.5047 (18)
Ni1—N31	2.1523 (11)	C33—H33	0.9500
Ni1—N41	2.1291 (11)	C33—C34	1.382 (2)
N1—C1	1.1643 (17)	C34—H34	0.9500
C1—S1	1.6254 (13)	C34—C35	1.3858 (19)
N2—C2	1.1546 (18)	C35—H35	0.9500
C2—S2	1.6366 (13)	C36—H36A	0.9800
N11—C11	1.3413 (16)	C36—H36B	0.9800
N11—C15	1.3437 (16)	C36—H36C	0.9800
C11—H11	0.9500	N41—C41	1.3409 (17)
C11—C12	1.3912 (17)	N41—C45	1.3423 (17)
C12—C13	1.3898 (19)	C41—H41	0.9500
C12—C16	1.5050 (18)	C41—C42	1.3894 (18)
C13—H13	0.9500	C42—C43	1.391 (2)
C13—C14	1.387 (2)	C42—C46	1.508 (2)
C14—H14	0.9500	C43—H43	0.9500
C14—C15	1.3855 (18)	C43—C44	1.380 (2)
C15—H15	0.9500	C44—H44	0.9500
C16—H16A	0.9800	C44—C45	1.3872 (19)
C16—H16B	0.9800	C45—H45	0.9500
C16—H16C	0.9800	C46—H46A	0.9800
N21—C21	1.3400 (16)	C46—H46B	0.9800
N21—C25	1.3436 (16)	C46—H46C	0.9800
C21—H21	0.9500	O1—C52	1.4195 (19)
C21—C22	1.3907 (18)	O1—C53	1.4202 (19)
C22—C23	1.3927 (19)	C51—H51A	0.9800
C22—C26	1.5045 (19)	C51—H51B	0.9800
C23—H23	0.9500	C51—H51C	0.9800
C23—C24	1.3841 (19)	C51—C52	1.497 (2)
C24—H24	0.9500	C52—H52A	0.9900
C24—C25	1.3864 (18)	C52—H52B	0.9900
C25—H25	0.9500	C53—H53A	0.9900
C26—H26A	0.9800	C53—H53B	0.9900
C26—H26B	0.9800	C53—C54	1.503 (2)
C26—H26C	0.9800	C54—H54A	0.9800
N31—C31	1.3452 (16)	C54—H54B	0.9800
N31—C35	1.3437 (17)	C54—H54C	0.9800
			
N1—Ni1—N2	178.45 (4)	N31—C31—H31	118.1
N1—Ni1—N11	89.58 (4)	N31—C31—C32	123.82 (12)
N1—Ni1—N21	90.33 (4)	C32—C31—H31	118.1
N1—Ni1—N31	91.33 (4)	C31—C32—C33	117.51 (12)
N1—Ni1—N41	89.18 (4)	C31—C32—C36	120.55 (12)
N2—Ni1—N11	89.02 (4)	C33—C32—C36	121.94 (12)
N2—Ni1—N21	90.29 (4)	C32—C33—H33	120.3
N2—Ni1—N31	90.07 (4)	C34—C33—C32	119.32 (12)
N2—Ni1—N41	90.18 (4)	C34—C33—H33	120.3
N11—Ni1—N31	179.05 (4)	C33—C34—H34	120.4
N21—Ni1—N11	88.35 (4)	C33—C34—C35	119.14 (13)
N21—Ni1—N31	91.93 (4)	C35—C34—H34	120.4
N21—Ni1—N41	178.94 (4)	N31—C35—C34	122.78 (12)
N41—Ni1—N11	90.72 (4)	N31—C35—H35	118.6
N41—Ni1—N31	89.02 (4)	C34—C35—H35	118.6
C1—N1—Ni1	153.43 (10)	C32—C36—H36A	109.5
N1—C1—S1	178.36 (12)	C32—C36—H36B	109.5
C2—N2—Ni1	159.93 (10)	C32—C36—H36C	109.5
N2—C2—S2	179.10 (13)	H36A—C36—H36B	109.5
C11—N11—Ni1	120.75 (8)	H36A—C36—H36C	109.5
C11—N11—C15	117.80 (11)	H36B—C36—H36C	109.5
C15—N11—Ni1	121.44 (8)	C41—N41—Ni1	121.14 (9)
N11—C11—H11	118.0	C41—N41—C45	117.88 (11)
N11—C11—C12	123.96 (12)	C45—N41—Ni1	120.95 (9)
C12—C11—H11	118.0	N41—C41—H41	118.1
C11—C12—C16	120.81 (12)	N41—C41—C42	123.82 (13)
C13—C12—C11	117.33 (12)	C42—C41—H41	118.1
C13—C12—C16	121.86 (12)	C41—C42—C43	117.17 (13)
C12—C13—H13	120.3	C41—C42—C46	121.06 (14)
C14—C13—C12	119.39 (12)	C43—C42—C46	121.76 (13)
C14—C13—H13	120.3	C42—C43—H43	120.1
C13—C14—H14	120.4	C44—C43—C42	119.85 (13)
C15—C14—C13	119.24 (12)	C44—C43—H43	120.1
C15—C14—H14	120.4	C43—C44—H44	120.6
N11—C15—C14	122.27 (12)	C43—C44—C45	118.89 (13)
N11—C15—H15	118.9	C45—C44—H44	120.6
C14—C15—H15	118.9	N41—C45—C44	122.38 (13)
C12—C16—H16A	109.5	N41—C45—H45	118.8
C12—C16—H16B	109.5	C44—C45—H45	118.8
C12—C16—H16C	109.5	C42—C46—H46A	109.5
H16A—C16—H16B	109.5	C42—C46—H46B	109.5
H16A—C16—H16C	109.5	C42—C46—H46C	109.5
H16B—C16—H16C	109.5	H46A—C46—H46B	109.5
C21—N21—Ni1	122.07 (8)	H46A—C46—H46C	109.5
C21—N21—C25	117.64 (11)	H46B—C46—H46C	109.5
C25—N21—Ni1	119.80 (8)	C52—O1—C53	113.17 (12)
N21—C21—H21	118.1	H51A—C51—H51B	109.5
N21—C21—C22	123.87 (12)	H51A—C51—H51C	109.5
C22—C21—H21	118.1	H51B—C51—H51C	109.5
C21—C22—C23	117.46 (12)	C52—C51—H51A	109.5
C21—C22—C26	120.49 (13)	C52—C51—H51B	109.5
C23—C22—C26	122.06 (12)	C52—C51—H51C	109.5
C22—C23—H23	120.3	O1—C52—C51	108.25 (12)
C24—C23—C22	119.38 (12)	O1—C52—H52A	110.0
C24—C23—H23	120.3	O1—C52—H52B	110.0
C23—C24—H24	120.5	C51—C52—H52A	110.0
C23—C24—C25	118.96 (12)	C51—C52—H52B	110.0
C25—C24—H24	120.5	H52A—C52—H52B	108.4
N21—C25—C24	122.64 (12)	O1—C53—H53A	110.0
N21—C25—H25	118.7	O1—C53—H53B	110.0
C24—C25—H25	118.7	O1—C53—C54	108.46 (13)
C22—C26—H26A	109.5	H53A—C53—H53B	108.4
C22—C26—H26B	109.5	C54—C53—H53A	110.0
C22—C26—H26C	109.5	C54—C53—H53B	110.0
H26A—C26—H26B	109.5	C53—C54—H54A	109.5
H26A—C26—H26C	109.5	C53—C54—H54B	109.5
H26B—C26—H26C	109.5	C53—C54—H54C	109.5
C31—N31—Ni1	121.89 (8)	H54A—C54—H54B	109.5
C35—N31—Ni1	120.64 (9)	H54A—C54—H54C	109.5
C35—N31—C31	117.39 (11)	H54B—C54—H54C	109.5
			
Ni1—N11—C11—C12	−179.71 (9)	C23—C24—C25—N21	0.0 (2)
Ni1—N11—C15—C14	179.93 (10)	C25—N21—C21—C22	1.80 (19)
Ni1—N21—C21—C22	−170.21 (10)	C26—C22—C23—C24	178.84 (14)
Ni1—N21—C25—C24	170.56 (10)	N31—C31—C32—C33	−1.89 (19)
Ni1—N31—C31—C32	−175.46 (9)	N31—C31—C32—C36	178.29 (12)
Ni1—N31—C35—C34	177.39 (10)	C31—N31—C35—C34	0.66 (19)
Ni1—N41—C41—C42	−177.75 (10)	C31—C32—C33—C34	0.66 (19)
Ni1—N41—C45—C44	177.81 (10)	C32—C33—C34—C35	1.1 (2)
N11—C11—C12—C13	−0.29 (19)	C33—C34—C35—N31	−1.8 (2)
N11—C11—C12—C16	−179.46 (12)	C35—N31—C31—C32	1.23 (19)
C11—N11—C15—C14	−0.57 (18)	C36—C32—C33—C34	−179.52 (13)
C11—C12—C13—C14	−0.44 (19)	N41—C41—C42—C43	−0.1 (2)
C12—C13—C14—C15	0.6 (2)	N41—C41—C42—C46	179.09 (14)
C13—C14—C15—N11	−0.1 (2)	C41—N41—C45—C44	−0.3 (2)
C15—N11—C11—C12	0.79 (18)	C41—C42—C43—C44	−0.2 (2)
C16—C12—C13—C14	178.71 (13)	C42—C43—C44—C45	0.2 (2)
N21—C21—C22—C23	−0.3 (2)	C43—C44—C45—N41	0.0 (2)
N21—C21—C22—C26	179.49 (14)	C45—N41—C41—C42	0.4 (2)
C21—N21—C25—C24	−1.63 (18)	C46—C42—C43—C44	−179.38 (15)
C21—C22—C23—C24	−1.4 (2)	C52—O1—C53—C54	174.86 (13)
C22—C23—C24—C25	1.5 (2)	C53—O1—C52—C51	176.55 (12)
