# Supplementary figures and images for: Syntheses and crystal structures of the ethanol, acetonitrile and diethyl ether Werner clathrates bis­(iso­thio­cyanato-κN)tetra­kis­(3-methyl­pyridine-κN)nickel(II)
Source: Acta Crystallogr E Crystallogr Commun. 2022 Sep 8;78(Pt 10):993–8. doi: 10.1107/S2056989022008891 (PMC9535821; doi:10.1107/S2056989022008891)

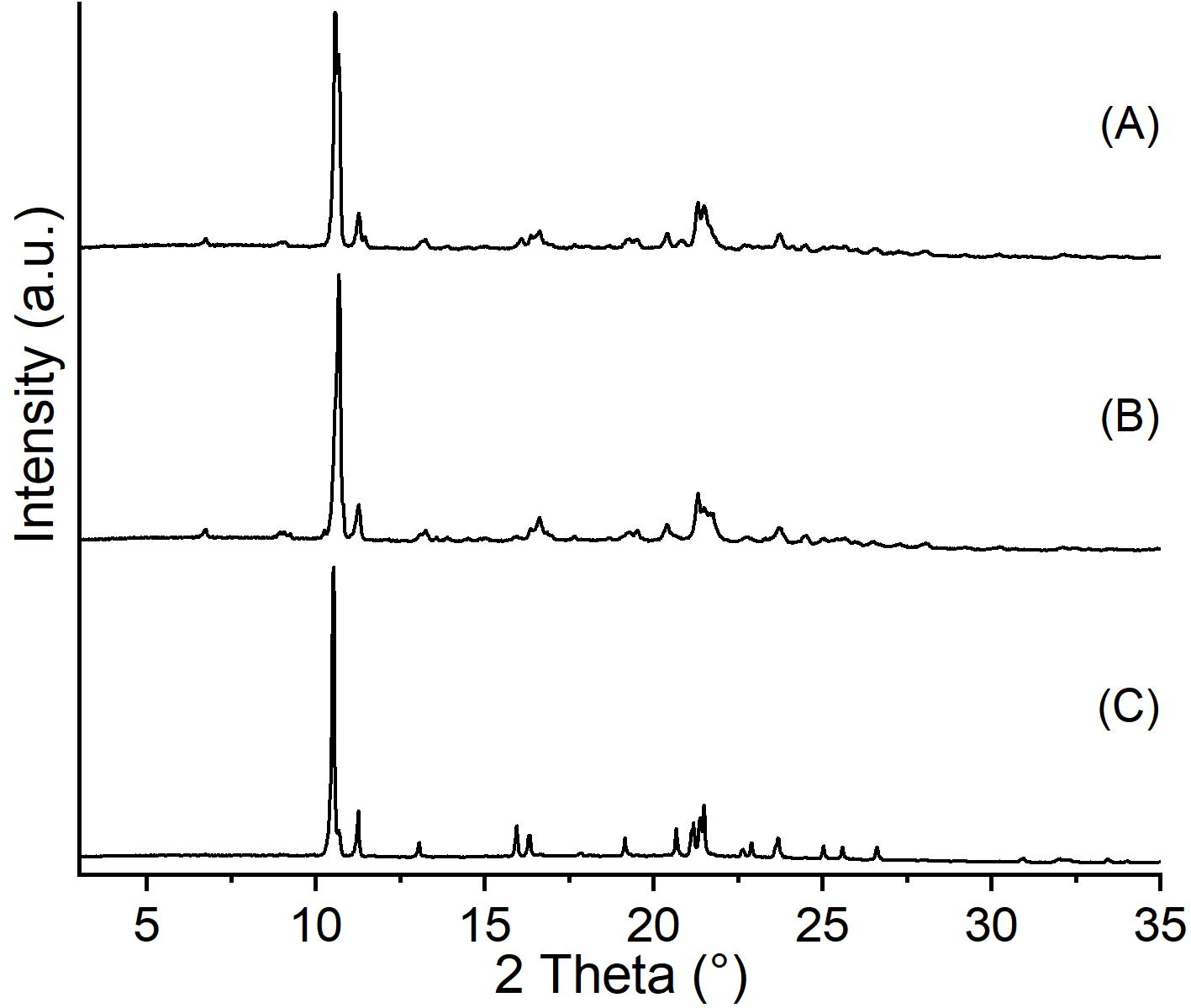

Supplement: Supplementary file 5 [file e-78-00993-sup5.png]

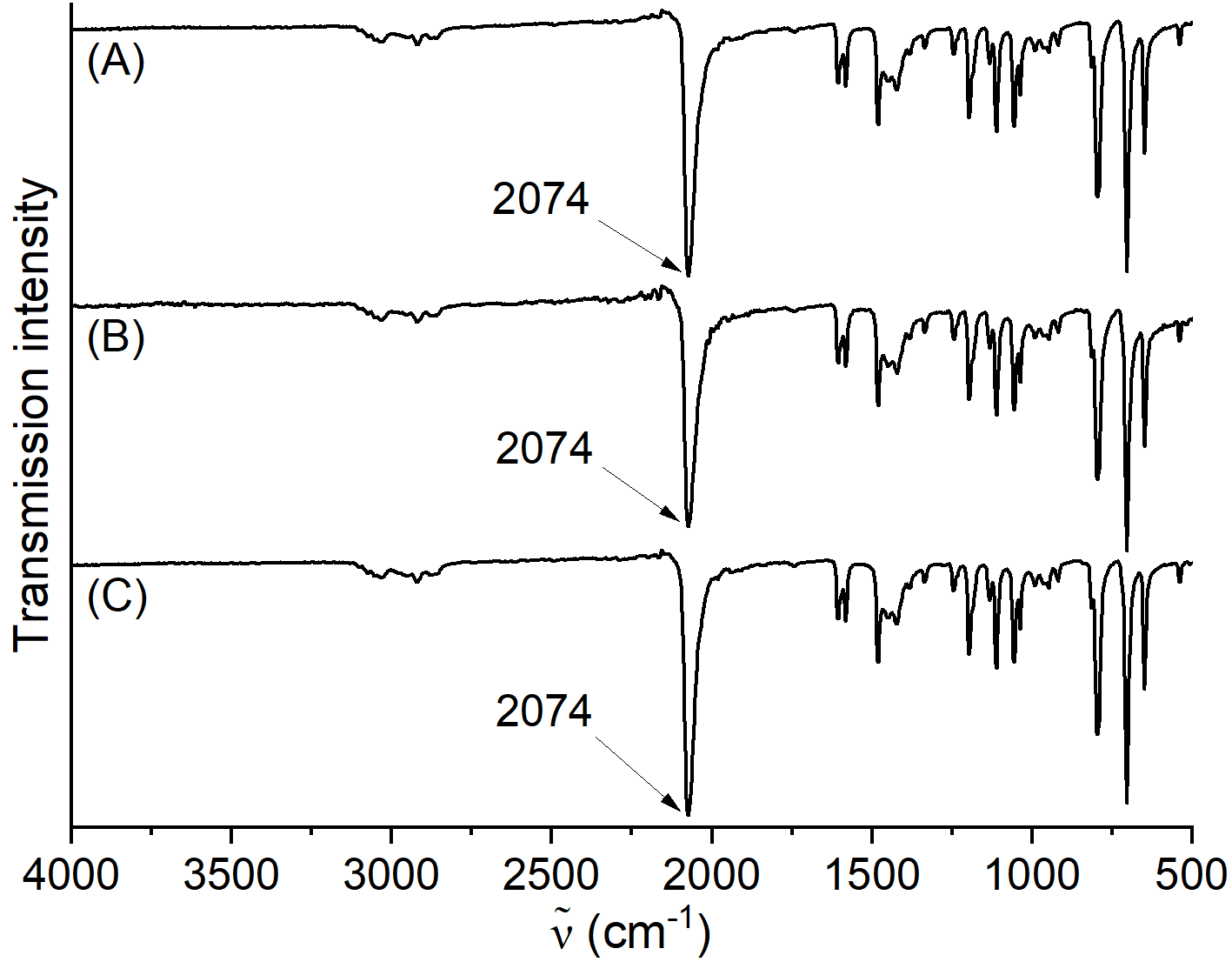

Supplement: Supplementary file 6 [file e-78-00993-sup6.png]
